# Supplementary material for: Wireless magneto-ionics: voltage control of magnetism by bipolar electrochemistry
Source: Nat Commun. 2023 Oct 14;14:6486. doi: 10.1038/s41467-023-42206-5 (PMC10576778; doi:10.1038/s41467-023-42206-5)
Supplement: Supplementary file 1 — Supplementary Information [file 41467_2023_42206_MOESM1_ESM.pdf]

Supplementary Information for

Wireless magneto-ionics: voltage control of magnetism by bipolar electrochemistry

Zheng Ma [1†], Laura Fuentes-Rodriguez [2,3†], Zhengwei Tan [1], Eva Pellicer [1], Llibertat Abad [3], Javier Herrero-Martín [4], Enric Menéndez [1*], Nieves Casañ-Pastor [2*] and Jordi Sort [1,5*]

[1] Departament de Física, Universitat Autònoma de Barcelona, Cerdanyola del Vallès, 08193, Spain

[2] Institut de Ciencia de Materials de Barcelona, CSIC, Campus UAB, 08193 Bellaterra, Barcelona, Spain

[3] Centre Nacional de Microelectrónica, Institut de Microelectrónica de Barcelona-CSIC, Campus UAB, 08193 Bellaterra, Barcelona, Spain

[4] ALBA Synchrotron Light Source, 08290 Cerdanyola del Vallès, Spain

[5] Institució Catalana de Recerca i Estudis Avançats (ICREA), Pg. Lluís Companys 23, Barcelona 08010, Spain

[†] These authors contributed equally to this work.

[*] e-mail: enric.menendez@uab.cat; nieves@icmab.es; jordi.sort@uab.cat

Table of Contents

Supplementary Session 1. Horizontal bipolar electrochemistry cells

Supplementary Session 2. Vertical BPE devices and the non-volatility of induced magneto-ionics

Supplementary Session 3. Linear sweep voltammetry measurements

Supplementary Session 4. Control experiments to rule out the formation of cobalt iodide under zero applied voltage

Supplementary Session 5. Finite element electrostatic modelling of the bipolar cells

Supplementary Session 6. Hysteresis loops of different zones of the sample treated in horizontal BPE

Supplementary Session 7. Magnetization reversibility tests for vertical BPE samples

Supplementary Session 8. N 1*s* X-ray photoelectron spectroscopy study for vertical BPE samples

Supplementary Session 9. Current profile for the connected Pt electrode circuit

References for Supplementary Information

Section 1. Horizontal bipolar electrochemistry cells

Representative horizontal bipolar electrochemical cells can be visualized in Fig. S1. After applying 10 V for 5 min, no appreciable signs of CoN reduction are observed on its cathodic pole (the right end of the sample in this case). Prolonging the voltage actuation time creates a few nitrogen bubbles or PC reduction products on the cathodic pole, as seen in Fig S1b-c (please note that the negative CoN pole is close to the external Pt positive pole). Significant changes occur when the driving voltage is further increased. Due to the creation of sufficiently large induced potential difference, the bubbling creeps towards the surface of the cathodic poles for bipolar electrochemical experiments above 10 V (Fig. S1d-e). Meanwhile, the bubbles get denser and denser in response to enhanced external voltages. Iodide, I^- oxidation to I_2 (or I_3^-) is observed at the wired Pt anode, and in smaller amount at the induced anode (left side of the sample) on the immersed unwired sample. During reduction the black CoN transforms into an apparently bright metallic cobalt (right side of sample).

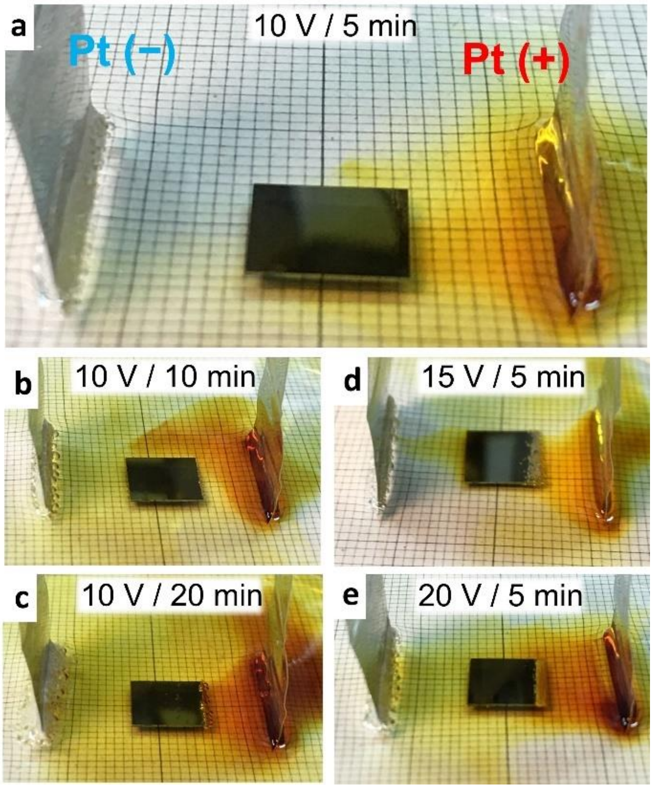

Fig S1 a-e, Representative optical images of the horizontal BPE cells. From **a** to **e**, they correspond to the BPE conditions of 10 V / 5 min, 10 V / 10 min, 10 V / 20 min, 15 V / 5 min, and 20 V / 5 min, respectively (The values before and after the slash symbols refer to the magnitudes of the driving voltage and application time, respectively). Note that graph papers were put underneath the glass cells to allow a reproducible arrangement of the sample and the electrodes.

Section 2. Vertical BPE devices and the non-volatility of induced magneto-ionics

Representative optical images showing the BPE device configuration and evolution of electrochemical/magneto-ionic processes are presented in Fig. S2. One can observe the electrochemical reactions on the driving electrodes (Fig. S2b and Fig S2c). While no bubbles on the CoN bipolar electrode are visible under 10 V, when the driving voltage increases to 15 V, small bubbles start to appear on the CoN BPE cathode pole in addition to the Pt driving cathode (see Fig. S2c). At this potential, the saturation magnetization (M_S) increases considerably (green curve in Fig. S2d). Further M_S increase is observed at 20 V. In addition, Fig 2e shows that consecutive hysteresis loops measurements (even after several hours from bipolar electrochemical experiments) do not reveal any degradation of the induced magnetic signal within the studied time intervals.

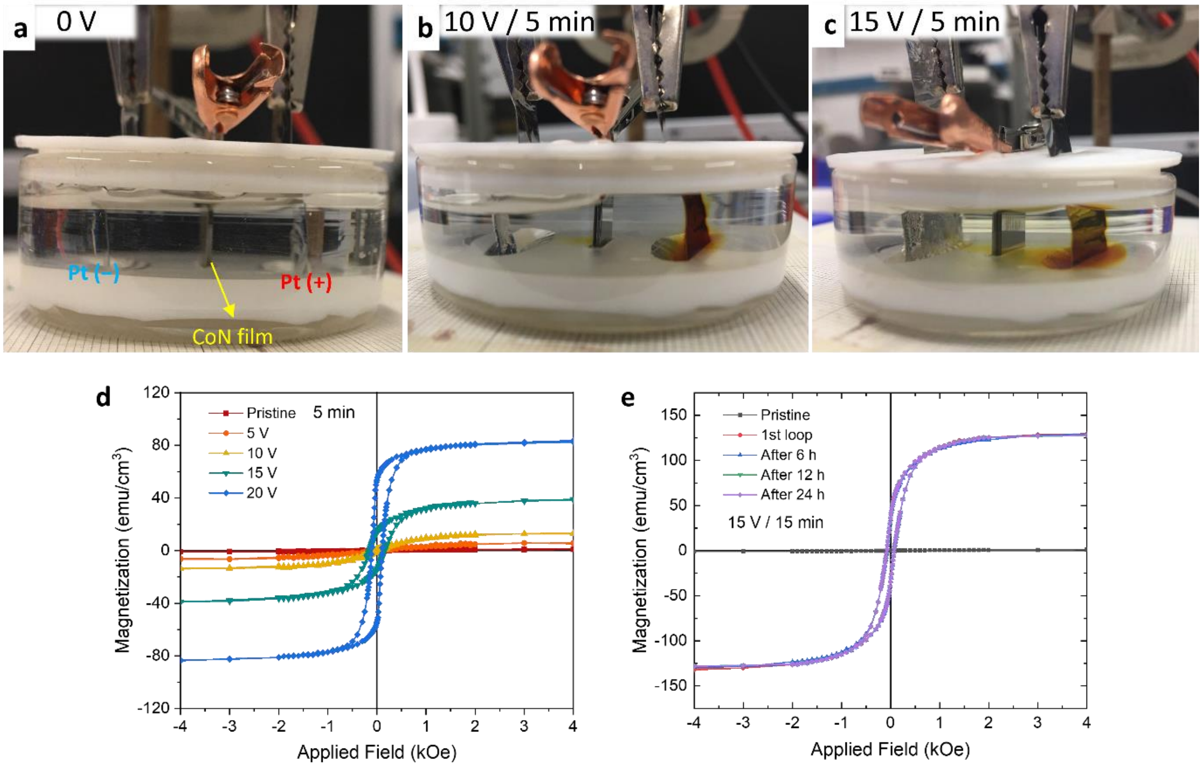

Fig. S2 a-c, Representative optical images of the vertical bipolar electrochemical cells. From **a** to **c**, they correspond to the initial state (no driving voltage applied), and application of external potential of 10 V / 5 min and at 15 V / 5 min, respectively. (The values before and after the slash symbols refer to the magnitudes of the driving voltage and application time, respectively). **d,** Room-temperature hysteresis loops at various driving voltages for 5 min actuation time. **e**, Room-temperature hysteresis loops for the as-prepared state and the sample treated at 15 V for 15 min. For the later, the loops were repeatedly recorded during >24 h (for clarity, only the first loop and the ones recorded after 6, 12 and 24 h are shown).

Table S1. Dependence of the coercivity and the squareness ration (M_R/M_S) on the driving voltage and actuation time.

	H_C (Oe)	M_R/M_S (%)
**15V 2.5min**	120	17.3
**15V 5min**	144	35.4
**15V 10min**	148	51.1
**15V 15min**	74	27.2
**15V 20min**	197	58.5
**5V 5min**	56	6.7
**10V 5min**	66	7.6
**15V 5min**	144	35.1
**20V 5min**	106	64.3
**5V 15min**	109	12.0
**10V 15min**	122	34.5
**15V 15min**	74	26.4
**20V 15min**	113	66.1

Section 3. Linear sweep voltammetry measurements

The possible redox processes on CoN ca n be studied by linear sweep voltammetry via direct contact of the sample. In this case, a three-electrode cell was used with a Pt sheet (15 mm × 35 mm in size) as a counter electrode, a Pt wire as a pseudo-reference electrode, and CoN as a working electrode. First, an attempt was made to characterize the electrochemical response of the PC electrolyte with 0.1M KI containing different amounts of water using a glassy carbon electrode 3 mm in diameter (Biologic) (Fig. S3a). Then, the electrochemical response of the CoN layer on Au/Ti/Si was studied and compared with the bare Au/Ti/Si. Linear scans were run from the rest potential down to –3 V vs Pt (Pt *vs*. Ag/AgCl approx. 0 V) with a speed of 5 mV s^{-1} (Fig. S3b).

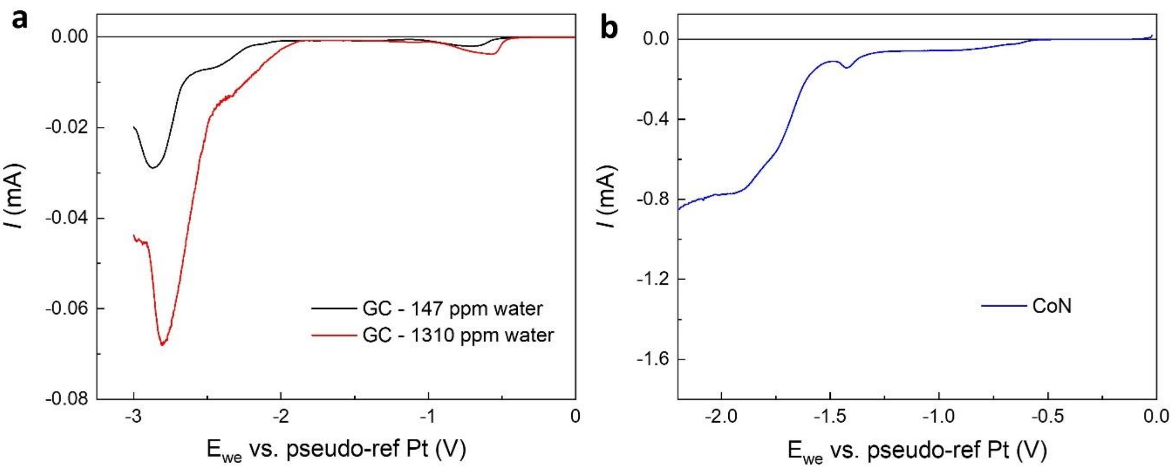

Fig. S3 a, Cathodic linear sweep voltammetry of PC with 0.1M KI and different water contents, recorded on a glassy carbon (GC) electrode vs. Pt. **b,** Cathodic linear sweep voltammetry of the CoN/Au/Ti/Si in a PC electrolyte with 0.1M KI. Note that no waves were observed or were very flat at potentials above 0 V vs reference.

The response of the electrolyte, using a glassy carbon as a working electrode, shows the presence of three reduction waves. The lowest intensity wave is at –0.7 V vs. Pt, followed by a wave at –2.42 V vs. Pt and, finally, a third wave of larger intensity at –2.86 V vs. Pt (see Fig. S3a). In principle, several reduction signals are expected for the electrolyte used: from the solvent PC itself, from iodine given the existence of KI that in an atmospheric medium could have formed I_2 (or I_3^-), and from H_2O dissolved by environmental absorption (its content determined by the Karl Fischer method is around 147 ppm). Subsequently, the same study was performed in a PC + 0.1 M KI solution with 10 times larger water content (red curve). The corresponding voltammetry curve shows an intensity enhancement for all the waves, evidencing that water plays a global role in the process, and is not a mere individual component. The largest relative enhancement is for the largest intensity wave at –2.8 V vs Pt. In addition, as water content increases, all the reduction waves are anodically displaced by 0.1 V, facilitating reduction processes. It is evident, therefore, that a greater presence of water substantially modifies the solvent and increases the ease of reduction (decreases the overpotential) in all processes.

Once the behavior of the electrolyte was established, the cathodic response of CoN on Au/Ti/Si was studied in PC with 0.1M of KI (Fig S3b). Three waves are observed, in addition to the –0.7 V vs Pt: the first at –1.42 V vs Pt, with lower intensity than the second, at –1.74 V vs Pt and the third –1.93 V vs Pt, the last two overlapping. Since both I_2 and water would be reduced at more positive potentials than CoN, it is considered that these last two waves correspond to the reduction of CoN.

Importantly, the underlying Au conducting layer does not contribute once the CoN is deposited on it. No clear reoxidation waves are observed for CoN reduction within the same potential window, suggesting that the reverse process may require an additional overpotential. The existence of two overlapping waves suggests that the reduction process of CoN could occur in several stages, but it also

evidences that the precursor layer of CoN could contain several stoichiometries, CoN_x, or several microstructures. This last possibility is in perfect agreement with the observations made by XPS that suggest the formation of two phases with different N/Co stoichiometry ratios.

Section 4. Control experiments to rule out the formation of cobalt iodide under zero applied voltage

Immersed CoN samples in the electrolyte for a period of 15 minutes show no change in saturation magnetization, M_S, as shown from the hysteresis loops in Fig S4a, evidencing the absence of a direct reaction of CoN either with I^- or I_2**,** implying that all observations reported correspond to the bipolar electrochemical treatment**.** The corresponding XPS survey shows no evidence of I 3d signals either.

Additional possible reactions were also discarded. Metallic cobalt formed during reduction at the bipolar electrode, does not react with I_2 formed at the driving anode electrode, as evident in Fig S4b, and despite thermodynamic expectations. To prove it, a large amount of pure cobalt powder was immersed in propylene carbonate (PC) containing I_2, and CoI_2 did not form within the first 48 hours (Figure S4b, left). When thiosulfate ($S_2O_3^{2-}$) was added to the vessel, the solution became uncoloured because I_2 is reduced back to I^-. If cobalt iodide would have formed, then the solution would become pink if some water is present or blue coloured in absence of water. However, this is not the case indeed. Figure S4b, right shows the uncoloured liquid after decantation to remove the cobalt powder.

a

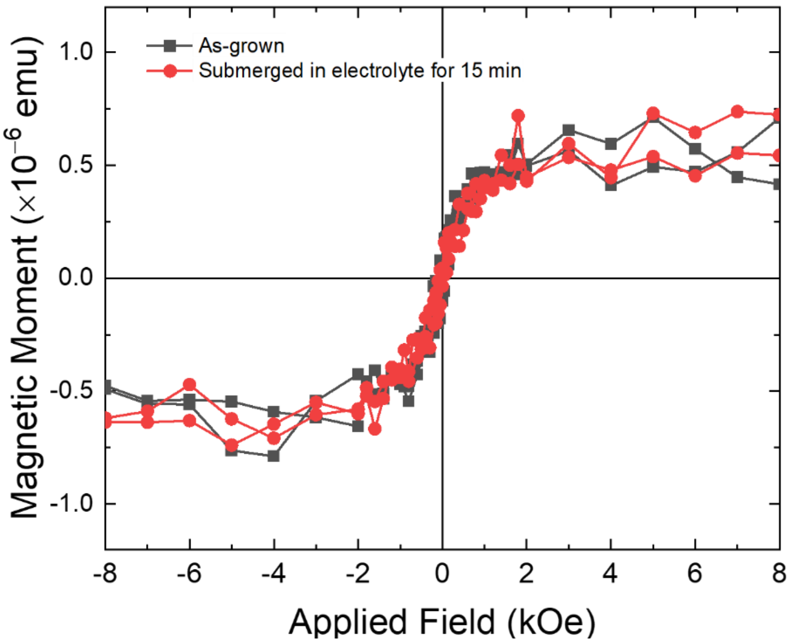

b

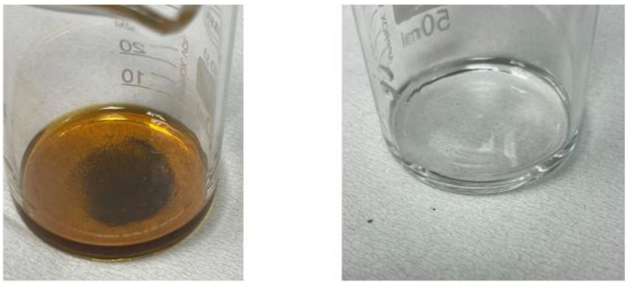

I_2 in PC + Co
No reaction

Solution resulting from:
$I_2 + Co + S_2O_3^{2-} \rightarrow 2I^- + 2SO_4^{2-} + Co$
NO pink or blue CoI_2 present

Re-reduction of I_2 to I^-
would allow to see CoI_2 if present (pink or blue)

Fig. S4 a) Room-temperature hysteresis loops for the as-grown CoN films with (in red) or without (in black) submerging in the liquid electrolyte. The submersion duration is 15 minutes. Note the low magnetic moment signal in the Y-axis for both cases. b) Cobalt powder in a solution of I_2 in PC, evidencing the absence of an spontaneous chemical reaction (left), and uncoloured liquid resulting from the addition of thiosulfate ($S_2O_3^{2-}$) to the previous vessel, which indicates that simply I_2 is reduced back to I^-. The liquid was decanted to remove the cobalt powder for better visualization.

Session 5 Finite element electrostatic modelling of the bipolar cells

Fig. S5 shows the distribution of induced potential *vs*. the electrolyte at the poles (*i.e.*, the edges of the magneto-ionic films), according to the electrostatic COMSOL simulations. For an external potential of 15 V in a horizontal geometry cell, *i.e*., the sample parallel to the applied field main axis, the induced potential is 7.2 V at the poles. On the other hand, for the vertical configuration and the sample perpendicular to the field axis, with an applied external voltage of 15 V, 1 V is obtained between poles. This simplified simulation shows that the dipole formed is smaller when the sample dimension along the field axis is reduced.

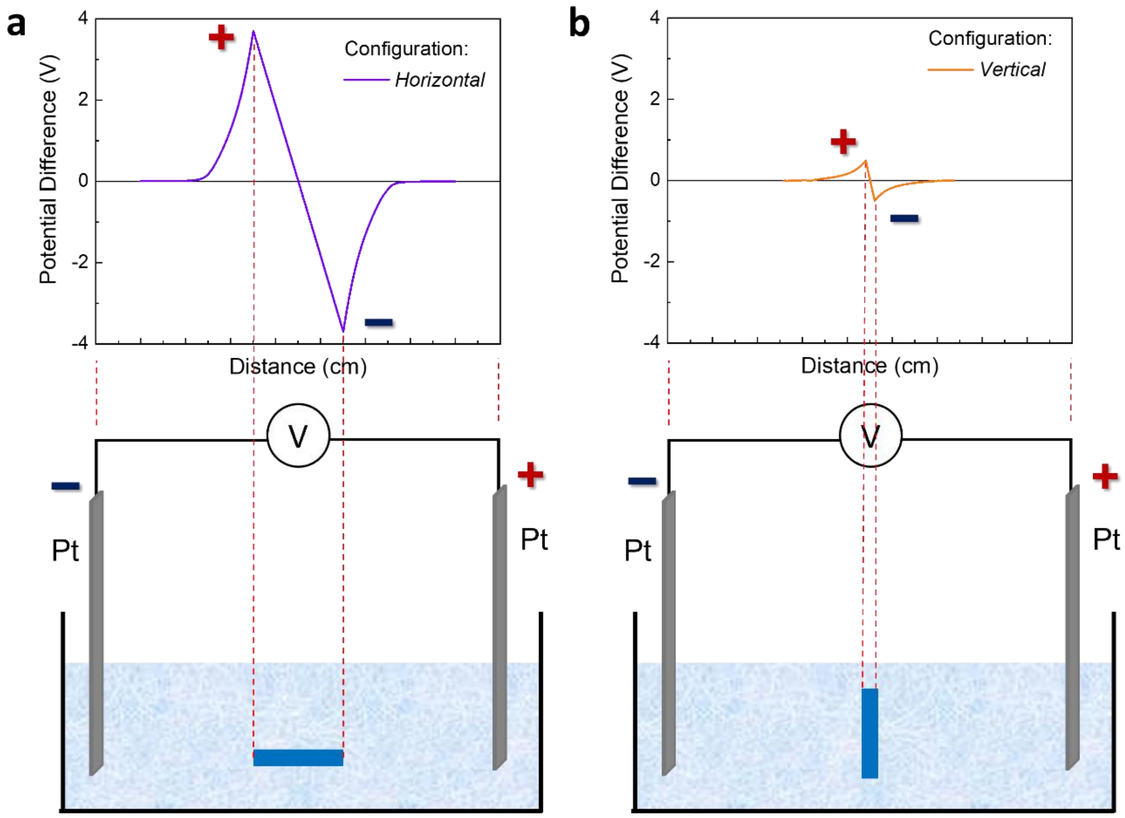

Fig. S5 COMSOL simulation of the potential distribution with respect to the electrolyte voltage when applying an external driving voltage of 15 V for the horizontal (a) and vertical (b) configurations. The same scale has been used for easy comparison. The red dashed vertical lines indicate the sample boundaries. Please note that the nm scale of the vertical configuration has been simplified to mm, since COMSOL cannot achieve such relative scales difference.

Section 6. Hysteresis loops of different zones of the sample treated in horizontal BPE

As a first estimation of the charge gradient in horizontally treated samples, we have measured the corresponding M_S signal for a sample split in three equal parts, the negative pole, the positive pole and the central part, immediately after the bipolar treatment. As seen in Fig. S6, the negative pole shows a

much larger M_S than that of the positive side, from measurements performed during the first hour after the treatment, which evidences a significant gradient, dynamic in nature. Thus, the magnetization enhancement is also observed to propagate towards the positive pole, either due to the bipolar treatment or the further relaxation. On the basis of these results, we hypothesize that, upon the removal of the external driving voltage, the charge gradient created along the sample and the corresponding redox and ionic changes promote internal diffusion of ions that equalize electronic oxidation states, resulting in an internal electrochemical discharge relaxation that restores electroneutrality.

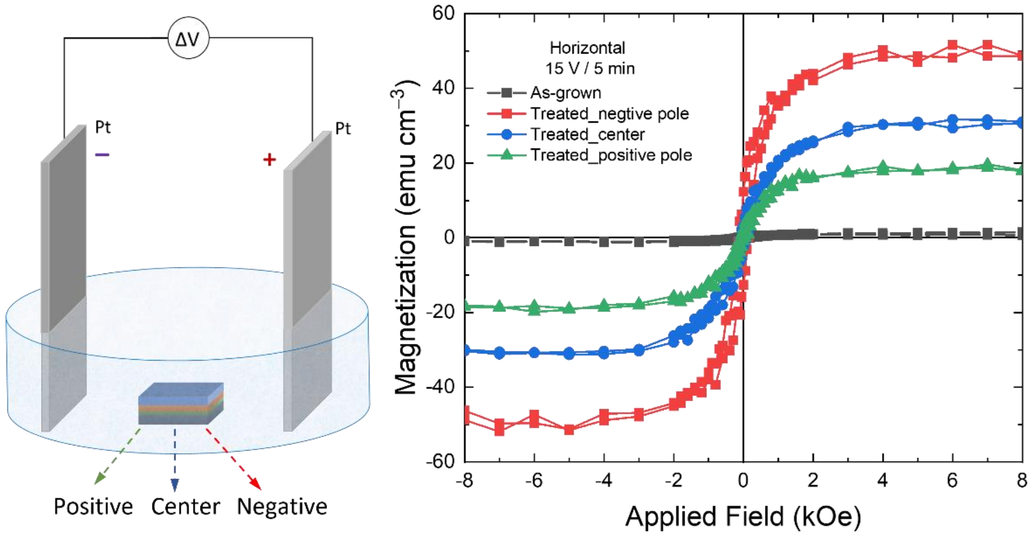

Fig. S6 *Ex situ* consecutive measurements of hysteresis loops corresponding to the negative and positive poles and the center of the sample, evidencing the redox gradient achieved after treatment. Note that recording each loop takes about 20 min. The figure on the left shows a schematic illustration of the sample in the cell.

Session 7. Magnetization reversibility tests for vertical BPE samples

Magnetic reversibility tests have been done in this configuration. For this purpose, a CoN film was actuated at an external driving voltage of 15 V for 5 min, giving rise to a M_S generation of 47.2 emu cm^{-3} (in red). Upon discharging the bipolar electrode and then reversing the driving voltage to −15 V, the M_S decreased considerably to 5.7 emu cm^{-3} (in blue), representing an 88 % drop in M_S. The remanent magnetic signal observed here may correspond to the extent in which nitrogen ions initially released to the solution are not able to be reinserted back to the sample. The chemistry and kinetics associated with this process needs further investigation, but it is important to point out that the wireless magnetoionic effect can be made largely reversible.

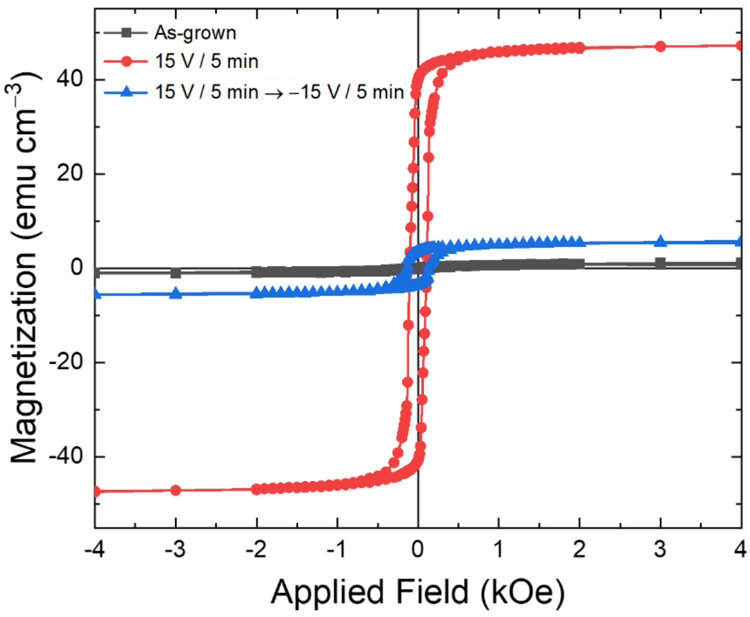

Fig S7 Room-temperature hysteresis loops for the as-grown (in black), treated (in red) and recovered (in blue) samples. The actuation is conducted under a condition of 15 V / 5 min, and the recovery is done firstly by discharging (ground) and then applying −15 V / 5 min (reversing the polarity of driving voltage). Both are done in the vertical BPE configuration.

Session 8. N 1*s* X-ray photoelectron spectroscopy study for vertical BPE samples

Fig. S8 shows a general survey spectra and N 1*s* XPS spectra for the as-grown film and the film treated at 15 V for 15 min in a vertical bipolar electrochemical cell. No Iodine I 3d $_{5/23/2}$ signals (615-630 eV) are observed in neither of the spectra measured. The corresponding N/Co atomic ratios are shown in Table S2. Both N 1s spectra have a peak maximum located at approximately 398 eV together with a small shoulder at slightly larger binding energy (≈ 400 eV). It has been reported that, in Co–N compounds, due to the charge transfer between Co and N atoms, the N 1*s* XPS peak tends to shift to lower binding energies as the nitrogen content increases[1]. Thus, we have deconvoluted the spectra assuming two different contributions or phases (**A** and **B**) of Co–N with dissimilar N/Co ratio. Phase **A**, which has a higher N/Co ratio, corresponds to the primary peak at 398 eV, and Phase **B**, with a lower N/Co ratio, would be contributing mainly to the shoulder peak at 399–400 eV (see Fig. S4). After identifying each phase, we have calculated the area of each component for the two spectra, and the results are listed in Table S2.

Table S2 Atomic ratio N/Co and Calculated areas of Phase **A** and Phase **B**, along with the ratios between the two areas, for the XPS core-level N 1s spectra of the as-grown film and the film actuated at 15 V for 15 min in vertical BPE cell.

	Atomic ratio N/Co	Area (Phase **A**)	Area (Phase **B**)	Area ratio (Phase **B**/**A**)
As-grown	0.11	3329	1620	48%
15 V/15 min	0.07	2658	1456	55%

While there is an overall N/Co atomic ration lower in the treated sample (0.07) than in the as prepared sample (0.11), there are different proportions of the N 1s signal within each sample. For the as-grown sample, the ratio between Phase **B** and Phase **A** is 48%, which is smaller than that for the treated sample (55%). These results indicate that there is a smaller fraction of Phase **A** and larger fraction of Phase **B** in the treated sample. Along with the decrease of the N/Co ratio, showing that the amount of nitrogen decreases upon voltage treatment, *i.e.*, cobalt becomes more reduced, in agreement with other characterization techniques, the comparison of phase **A** and **B** implies that the decrease corresponds mostly to phase **A**.

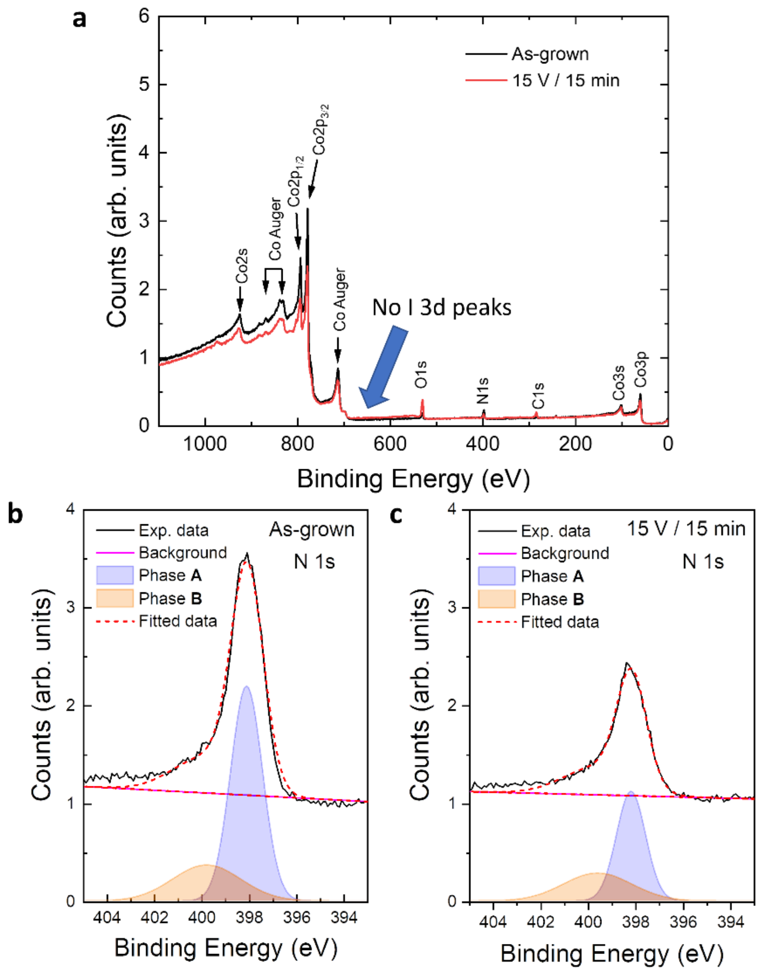

Fig. S8 ***a***, General survey spectra for the as-grown sample and treated sample at a driving voltage of 15 V for 15 min. The N 1*s* X-ray photoelectron spectra for the as-grown sample, **b**, and treated sample at a driving voltage of 15 V for 15 min, **c**. Spectra have been fitted considering the presence of two Co-N phases in both samples: "Phase **A**" (in bluish), which has a higher N/Co ratio at lower binding energy and "Phase **B**" (in brown) with a lower N/Co ratio at higher binding energy. Solid black lines correspond to experimental data, whereas dash-dotted red lines refer to the fitted data.

Section 9. Current profile for the connected Pt electrode circuit

The current observed through the connected Pt electrodes during the experiments always followed an exponential decrease, as expected in any electrochemical process. I^- and solvent reactions occur at the driving electrodes, and the extent of the reaction decreases with time. Please note that the processes occurring at the unwired bipolar electrode are not reflected in these curves, and that, by intrinsic definition, those currents cannot be registered through a wired connection.

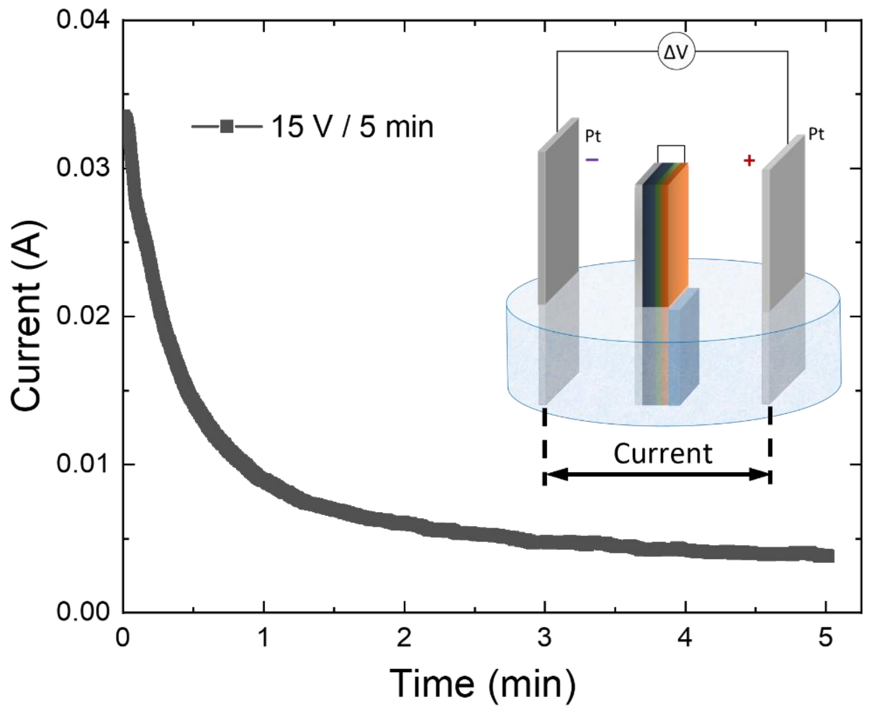

Fig. S9 Representative Current versus time plot, through the Pt connected driving electrodes during the bipolar experiment.

References

1. De La Cruz, W., Contreras, O., Soto, G. & Perez-Tijerina, E. Cobalt nitride films produced by reactive pulsed laser deposition. *Rev. Mex. Fis.* **52**, 409–412 (2006).
